# Pulsatile Drug Delivery System Triggered by Acoustic Radiation Force

**DOI:** 10.3389/fbioe.2020.00317

**Published:** 2020-04-17

**Authors:** Sabrina Ciancia, Andrea Cafarelli, Anna Zahoranova, Arianna Menciassi, Leonardo Ricotti

**Affiliations:** ^1^The BioRobotics Institute, Sant’Anna School of Advanced Studies, Pisa, Italy; ^2^Departments of Excellence, Robotics & AI, Sant’Anna School of Advanced Studies, Pisa, Italy; ^3^Department for Biomaterials Research, Polymer Institute SAS, Bratislava, Slovakia

**Keywords:** acoustic radiation force, ultrasound, chronotherapy, pulsatile drug delivery, controlled drug release, targeted therapy

## Abstract

Since biological systems exhibit a circadian rhythm (24-hour cycle), they are susceptible to the timing of drug administration. Indeed, several disorders require a therapy that synchronizes with the onset of symptoms. A targeted therapy with spatially and temporally precise controlled drug release can guarantee a considerable gain in terms of efficacy and safety of the treatment compared to traditional pharmacological methods, especially for chronotherapeutic disorders. This paper presents a proof of concept of an innovative pulsatile drug delivery system remotely triggered by the acoustic radiation force of ultrasound. The device consists of a case, in which a drug-loaded gel can be embedded, and a sliding top that can be moved on demand by the application of an acoustic stimulus, thus enabling drug release. Results demonstrate for the first time that ultrasound acoustic radiation force (up to 0.1 N) can be used for an efficient pulsatile drug delivery (up to 20 μg of drug released for each shot).

## Introduction

Nowadays, interest in novel drug delivery technologies is increasing. Innovation in this field would enable us to overcome the problems currently affecting conventional drug administration methods (e.g., oral, rectal, subcutaneous, intravenous or intramuscular ones). Indeed, such methods are not always effective and do not allow us to keep the drug dose inside the required and often narrow therapeutic window (Timko et al., 2010).

Targeted therapies aim to minimize side effects and may allow to release *in situ* a well-controlled quantity of drug, thus resulting in an improved therapeutic efficacy and a reduced systemic toxicity (Ricotti et al., 2015). In recent years, both spatially and temporally controlled drug delivery systems (DDS) have been developed. Spatially controlled DDS are based on a relatively high sensitivity to endogenous chemico-physical conditions (e.g., changes in pH, enzyme concentration, or redox gradients).

Certain environmental changes are specific of a particular pathological condition which can thus trigger drug release *in situ* (Mura et al., 2013).

In addition, temporal control of drug release can be achieved by making DDS responsive to exogenous (remote) stimuli [e.g., magnetic or electric fields, light or ultrasound (Wang and Kohane, 2017)], thus to precisely determine the timing, duration and dosage, besides the location of drug release. This kind of system could be exploited for pulsatile drug delivery, which consists of rapid and transient releases of a certain amount of drug molecules (Jain et al., 2011). A pulsatile release is based on a repeated succession of pulses at variable time intervals in coordination with a remote stimulation, which can deliver *in situ* a drug at pre-determined time-points.

Pulsatile Drug Delivery Systems (PDDS) are therefore designed to release the drug in the right site, at the right time and in the right amount according to the circadian rhythm of the human body. Indeed, humans exhibit a circadian rhythm (24-hour cycle) that occurs in several physiological processes and is regulated by the suprachiasmatic nucleus, which is located at the base of the hypothalamus (Khan et al., 2009). Thus, coordination between biological rhythms and medical treatment could provide maximum health benefits and minimum harm to the patient.

This medical treatment approach is known as “chronotherapy.”

The potential benefits of chronotherapy have been demonstrated for several chronotherapeutic disorders that are characterized by the onset of symptoms at given times of the day. These diseases follow a biological rhythm and they affect cardiovascular, gastrointestinal, respiratory and skeletal systems, inflammatory processes and neoplasms (Vipul and Moinuddin, 2012) (e.g., peptic ulcer, cancer or arthritis).

In addition to chronotherapeutic treatments, PDDS can be also used whenever a multiple dosing is required for a single implant.

As mentioned, a pulsatile drug release can be achieved by exploiting external and wireless physical energy sources.

The focus of this paper is on PDDS triggered by ultrasound (US), which are pressure waves with frequencies higher than 20 kHz, widely used for diagnosis (Shung, 2015) and therapeutic purposes (Escoffre and Bouakaz, 2015).

Therapeutic US can be targeted toward specific tissues and cells in a fully non-invasive manner. US are in fact non-ionizing radiations, which can penetrate safely and deeply into the body by tuning stimulation parameters (i.e., frequency, intensity, duty cycle, and exposure time).

Thermal and/or mechanical effects of US are already widely exploited both for one-shot drug delivery systems and for pulsatile release systems. Thermal effects consist of a temperature increase caused by the absorption of acoustic energy in tissues and they are exploited both to enhance drug uptake of targeted cells and as a trigger to release the drug from thermosensitive vectors.

Mechanical effects include acoustic cavitation, acoustic streaming and radiation force. The most straightforward method to deliver genes, proteins or smaller molecules by US is the use of microbubbles (Kooiman et al., 2010) that enhance the extravasation and cellular uptake of these compounds.

They can be also used to disrupt the structure of the drug carrier, thus enabling a target drug delivery (Pitt et al., 2004; Sennoga et al., 2017). However, the microbubbles must be injected in the bloodstream and they have limited stability *in vivo*, thus reducing the storage efficiency. Other examples of micro- and nano-carriers, such as micelles and liposomes, have been exploited for US-triggered continuous drug release (Abdel-Hafez and Husseini, 2015; Klibanov and Hossack, 2015; Ricotti et al., 2015; Chiu et al., 2017).

Although these carriers can be functionalized to recognize specific sites, thus limiting non-specific drug accumulation and reducing side effects, there are currently several issues (e.g., low mechanical stability, small size, low drug encapsulation capacity and slow extravasation) that are limiting their use in the clinics (Yokoyama, 2011).

In addition to the vectors mentioned above, there are some other studies about hydrogels opportunely functionalized to be reversibly responsive to US, and thus used as US-triggered pulsatile drug delivery. Several works on hydrogels with a reversible crosslinking are reported (Huebsch et al., 2014; Huang et al., 2017) where the hydrogel network was temporarily disrupted upon mechanical stimulation through US, thus enabling the drug release. Once the stimulus was removed, the polymer network self-healed again and restored its shape. Kwok et al. (2000) developed a polymer network/hydrogel, coated with an US-responsive self-assembled monolayer, based on ordered alkyl chains. Without the US-stimulation, this coating acts as a barrier to prevent the release of the drug from the hydrogel. During the stimulation, the coating is reversibly disrupted, enabling the drug to be released (Ruegsegger et al., 2001).

Ordeig et al. (2016) proposed a thermoresponsive hydrogel able to reversibly release the encapsulated drug after an overheating produced by US.

Vannozzi et al. (2016) proposed a US-responsive multilayer ultra-thin film based on poly(L-lactic acid) (PLLA) and polyelectrolytes. Responsivity to US was endowed by doping the PLLA layer with piezoelectric BaTiO3 nanoparticles.

When high targeting spatial resolution is required, focused US beams are preferably used. Recently, Moncion et al. (2018) studied the possibility to use focused US for a spatiotemporally controlled release of two different growth factors from an acoustically responsive scaffold, in order to help angiogenesis and osteogenesis. Morse et al. (2017) studied the effect of focused US on microbubbles in order to locally and noninvasively open the blood-brain barrier. They evaluated the ability of a rapid short-pulse sequence to improve the *in vivo* performance and safety of ultrasound-mediated drug delivery to the brain.

Di et al. (2014, 2017) instead, proposed a spatiotemporally controlled insulin delivery system consisting of an injectable polymeric nanoparticle-cross-linked network which was noninvasively triggered by a focused ultrasound system.

All the reported works on US-triggerable hydrogels rely on multistep and time-consuming hydrogel preparation, often requiring complicated chemical syntheses and purification steps. To avoid these issues, we developed a device responsive to acoustic radiation force, in which a hydrogel embedded with the desired drug can be placed, without any need of chemical modifications. Albeit in literature there are some works about PDDS triggered by US, no one reports the use of the acoustic radiation force as a trigger for drug release. So far, focused US has been used for thermal ablation at high intensities (> 100*W*/*cm*^2^) and only more recently for targeted drug delivery at low intensities (< 1*W*/*cm*^2^) (Ordeig et al., 2016; Moncion et al., 2018).

The use of a focused US beam guarantees a high spatial resolution.

In this paper we present a proof of concept of a PDDS remotely triggered by the acoustic radiation force of US, which is largely unexplored in the field of drug release (Lum et al., 2006) as a trigger mechanism. The proposed strategy has the potential to avoid harmful thermal or cavitational effects and to overcome the key limitations of the previously described state of the art solutions.

**FIGURE 1:**
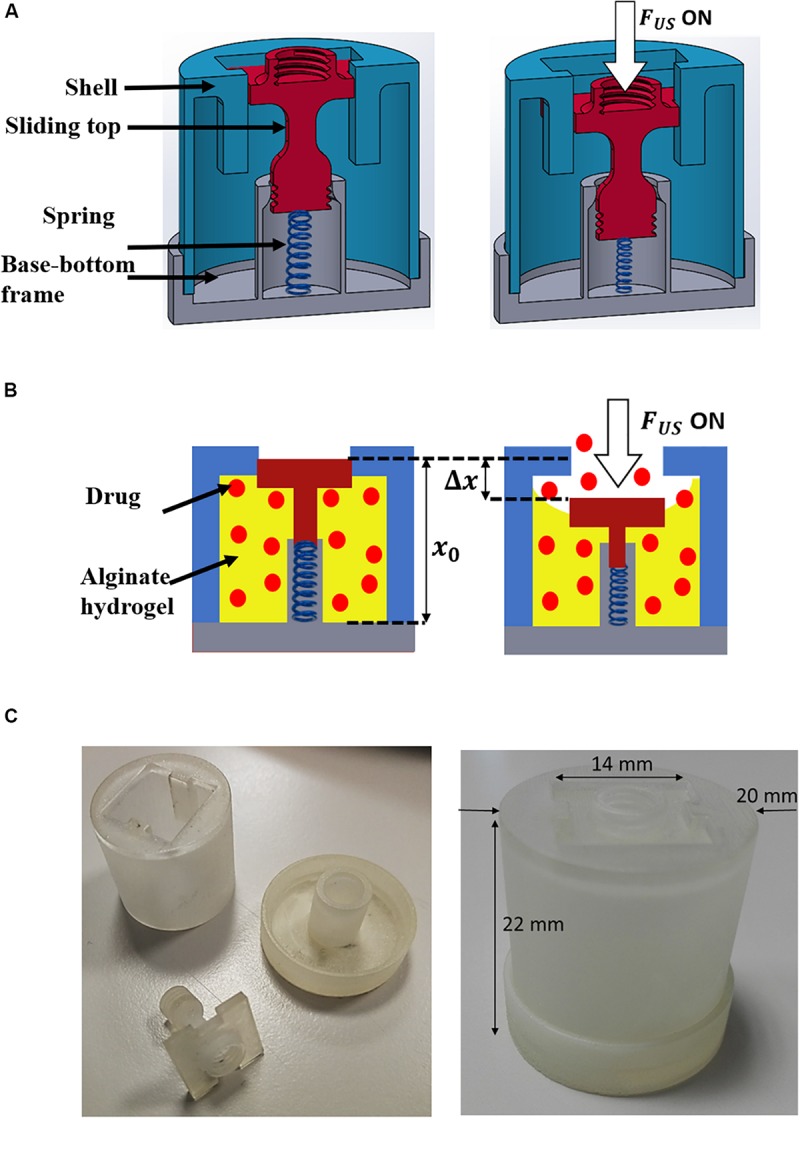
Depiction of the device and scheme of the concept: **(A)** CAD of the device cross-section in the OFF (left) and ON (right) configurations. ON corresponds to the application of US. **(B)** Scheme of the device working principle: The alginate gel is shown in yellow; the drug molecules are represented as red spots. **(C)** Picture of the device in a plastic material obtained by using a 3D Printer.

The device is shown in Figure 1 and consists of a case in which a gel loaded with the drug can be trapped and a sliding top that can be moved by the application of an acoustic stimulus, enabling an “on-demand” drug regulation, and controlling time, site and dosage of the drug delivered.

In the following sections the approach followed to design the system, the process to prepare and to characterize the alginate hydrogels are described. We selected the alginate-based hydrogel because it is widely used in literature for drug delivery devices, due to its high biocompatibility. In addition, the process through which the drug model is encapsulated in this hydrogel avoids washing steps that could lead to drug losses (such washes are not needed because no toxic compounds are needed to promote gelation). Further, system modeling was carried out by means of finite element model tools, in order to find the optimal value of the spring stiffness allowing a proper displacement of the sliding top. Then, the acoustic radiation force was measured and parameters such as power and duty cycle of the input wave were carefully selected to enable the proper displacement of the sliding top while avoiding local over-heating. Drug delivery tests on the device subjected to the acoustic radiation force are then reported. These tests demonstrated the possibility to release drug doses at specific time points by externally triggering the delivery in a non-invasive manner. Finally, further *in vitro* experiments were carried out by using a mimicking tissue phantom, in order to have a more convincing demonstration of the proposed technology.

## Concept and System Design

As anticipated, the proposed PDDS exploits the acoustic radiation force, which is a phenomenon involving any electromagnetic or acoustic waves. The waves exert a pressure on the bodies on the path, due to the momentum transfer from the waves to the matter. The acoustic radiation force is produced by a change in the energy density and momentum of the propagating waves caused by absorption, scattering or reflection phenomena (Sarvazyan et al., 2010). In terms of equations the acoustic radiation force *F*_*US*_ can be defined as follows:

(1) $$F_{U\,S}=2\,\alpha \,\frac{I}{c_{0}}$$

where α is the absorption coefficient, *c*_*0*_ is the equilibrium speed of sound in the medium and I is the intensity of the acoustic wave.

The hypothesis behind the proposed system is that acoustic radiation force can be used to push the top of the device case against an alginate hydrogel previously reticulated inside the device (Figure 1A). The drug entrapped within the hydrogel can therefore be squeezed through the lateral openings (Figure 1B). The top is linked to the base of the device through a spring properly dimensioned to allow a sufficient displacement of the top when the radiation force is applied, and its repositioning when the stimulus stops.

The device has been designed by using Solidworks (3D CAD design software, Dassault Systèmes) and printed (Figure 1C) by using a plastic material (Visijet M3 Crystal) through a 3D printer (ProJet MJP 3600 Series, 3D Systems). A preliminary analysis of the balance of acting forces was performed. The forces involved at equilibrium are: the acoustic radiation force (*F*_*US*_), the compressive force of the gel (*F*_*gel*_), the elastic force of the spring (*F*_*s*_) and the friction between guides and the top.

Compression tests carried out on the device without the gel inside by using an INSTRON 4464 Mechanical Testing System allowed us to verify that the friction between the sliding top and the guides is negligible in the system with respect to the acoustic radiation force (as shown in Supplementary Figure S2). Thus, the balance equation can be expressed as:

(2) $$F_{U\,S}=F_{g\,e\,l}+F_{s}$$

Once the radiation force is applied perpendicularly to the top, the gel undergoes a displacement (Δ*x*)along the direction of the applied force, starting from the initial position (*x*_*0*_).

Considering the elastic compressive modulus of the gel (*E*_*C*_), the area of the gel pressed by the top (*A*), and the Poisson Modulus (ν), the force exerted by the gel is the following:

(3) $$F_{\mathrm{gel}}={\mathrm{AE}}_{c}\,\frac{\Delta \,x}{x_{0}}\,\frac{1}{\nu }$$

The force of the spring is defined by Hooke’s law:

(4) $$F_{s}=k\,\Delta \,x$$

Where *k* is the spring stiffness.

Substituting (3) and (4) in (2), the displacement of the top can be obtained as follows:

(5) $$\Delta =\frac{F_{\mathrm{US}}}{k+\frac{{\mathrm{AE}}_{c}}{x_{0}\,\nu }}$$

### Preparation of Alginate Hydrogels by Internal Gelling Method

Sodium alginate with high content of G-monomer units (Protanal LF 10/60, FMC BioPolymer) was used to prepare alginate hydrogels, by internal gelling method (Draget et al., 1990; Papajová et al., 2012).

**FIGURE 2:**
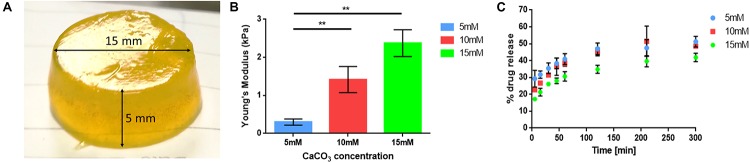
**(A)** Picture of an alginate gel prepared by internal gelling method, to be used for compression tests and drug release tests. **(B)** Histograms of the gel Young’s moduli in correspondence to different CaCO3 concentrations (5, 10, and 15 mM) (*n* = 6). **(C)** Drug release curves of the 5, 10, and 15 mM alginate gels (*n* = 6). **p < 0.01.

Briefly, three different gelling solutions were prepared by mixing an alginate stock solution (2.2 wt%) with stock solutions of CaCO_3_ in 0.9% NaCl at different concentrations. Fluorescein sodium salt in a 0.9 wt% NaCl solution, was used as drug model and added to the mixtures. Subsequently, D-(+)-Glucono- δ-lactone (GDL) in 0.9 wt% NaCl solution was quickly added to decrease the pH to about 6.5. The final concentration of the components in the mixture were 2 wt% of sodium alginate, 60 mM of GDL, 1 mg/mL of fluorescein sodium salt and three different concentrations of CaCO_3_ (5, 10, and 15 mM). The mixtures were pipetted in a 24 well plate and then maintained at 5–6°C for at least 24 h to allow gelation. Finally, cylindrical gels with a diameter of 15 mm and a height of about 5 mm were obtained (Figure 2A). The volumes and dimensions of the gels differ depending on the final application. For the pulsatile drug delivery tests the gels were directly produced inside the device.

### Compression Tests on Hydrogels

The compressive elastic modulus of the gels was tested by using the INSTRON 4464 Mechanical Testing System provided with a 10 N load cell. The compression tests were performed at a velocity of 5 mm/min and the data were acquired at a frequency of 100 Hz.

For each CaCO_3_ concentration, 7 samples were tested. The results are shown in Figure 2B in terms of mean values ± standard error of the mean. A Holm-Sidak test was performed for comparison between two groups. Significance was set at 5%.

Results highlighted a clear Young’s modulus increase with increasing CaCO_3_ concentrations (0.3 ± 0.1 kPa for 5 mM, 1.4 ± 0.34 kPa for 10 mM, 2.4 ± 0.35 kPa for 15 mM).

### *In vitro* Drug Delivery Tests on Internally Gelled Hydrogel

In order to test the gel release kinetics, drug release from the different gel samples was measured over 5 h. Release tests were performed in 2 mL of 0.9 wt% NaCl solution. At selected time points (5, 15, 30, and 45 min, 1, 2, 3.5, and 5 h) the fluorescence intensity was measured at excitation 485 nm – emission 535 nm, using a Plate Reader (VICTOR X3, PerkinElmer).

Results (Figure 2C) showed an inverse correlation between the concentration of crosslinker (CaCO_3_) and the drug release from the gels. Indeed, softer gels (5 mM) had slightly faster drug release dynamics with respect to the stiffer ones (10 and 15 mM).

In order to achieve the maximum displacement of the device top and to have the maximum drug release, the softer gel (5 mM) was selected for final drug delivery tests.

### FEM Simulations

In order to test the dynamic behavior of the proposed device and to select the optimal spring stiffness accordingly, the system was modeled by means of finite element analysis (FEM) simulations, through Abaqus 6.13 (Dassault Systèmes).

First, the device model was generated, consisting of: (i) a 22 × 20 mm cylinder mimicking the gel, with an internal hollow opening (diameter: 3 mm), accounting for spring positioning, (ii) a 22 × 22 mm shell, (iii) a 14 × 14 × 2 mm sliding top, and (iv) a spring attached to the sliding top through a cylindrical link of 2.8 × 10 mm. The mesh size was set at 0.6 mm for the sliding top and the alginate gel (set as solids in the simulation) and 0.8 mm for the shell.

The gel density was calculated as the ratio between sample mass and volume. The volume was measured by using a water-displacement technique as reported in the study of Prokop et al. (2003), and the mass was measured by weighting the samples. The measured gel density resulted in 950 kg/mm^3^. This value was set in the simulation by considering the alginate gel as an elastic isotropic material and assuming a uniform distribution of its density. The Poisson’s ratio was set equal to 0.4 (Chippada, 2010) and the Young’s modulus was set to 0.3 kPa in agreement with the experimental mechanical tests shown in see section “Compression Tests on Hydrogels.”

The contact pairs interaction was used to define contact constraints between surfaces. The contact property options between the surfaces generated (sliding top/alginate gel and alginate gel/shell) were assumed as frictionless (as concerns the tangential behavior) and hard contact (as concerns the normal behavior).

Concerning mechanical constraints, the sliding top and the superior and lateral surfaces of the alginate gel were allowed to translate along *Z*-axis. All other surfaces (i.e., shell and the bottom surface of the alginate gel) were blocked in their translations and rotations.

**FIGURE 3:**
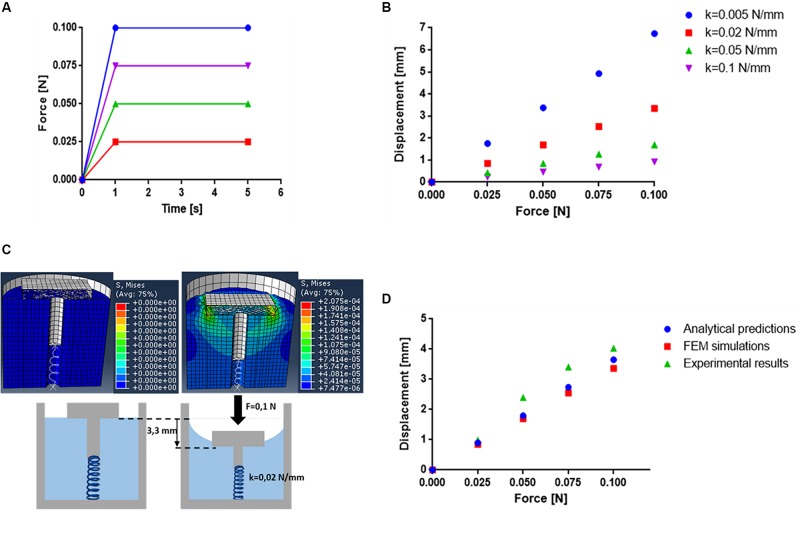
**(A)** Forces applied on the sliding top during the simulation on Abaqus. **(B)** Results obtained from FEM simulations in terms of displacement of the sliding top following the application of 4 different forces (0.025, 0.05, 0.075, and 0.1 N) and with 4 different spring’s stiffness (0.005, 0.02, 0.05, and 0.1 N/mm). **(C)** Frames of the simulation on Abaqus with values of pressures exerted on the top. **(D)** Graphs of the force applied on the sliding top of the device in function of its displacement: analytical results (blue), simulation results (red) and compression tests results (green) (*n* = 10).

Four different forces (0.025, 0.05, 0.075, and 0.1 N) were applied perpendicularly to the sliding top (Figure 3A). This range was considered a reasonable one to be achieved by acoustic radiation force (see section “Discussion”). Four different spring stiffness levels (0.005, 0.02, 0.05, and 0.1 N/mm) were tested for each value of force applied to the sliding top and the top vertical displacement was evaluated, as simulation result (Figure 3B and Supplementary Movie S1).

The spring’s stiffness of 0.02 N/mm was selected for drug delivery tests because it allows a considerable displacement of the top (up to ∼4 mm) with the imposed forces; this value is sufficient to leave the internal environment in communication with the external one and thus to allow drug release. This value of spring’s stiffness is also able to counteract the friction of the sliding top on the guides allowing a good elastic return after removal of the stimulus, as demonstrated in the results shown in the next sections.

In addition, FEM simulations (Figure 3C) revealed that also in the case of the maximum force imposed (0.1 N), the maximum stress exerted on the gel was 0.2075 kPa, which is lower than the ultimate strength of the gel [(1.50 ± 0.12) kPa]. Thus, in this configuration, the system is efficiently compressed without compromising its structural integrity.

Results (Figures 3B,C) highlighted a good agreement between analytical predictions, simulations and experimental data.

In order to validate the simulation results, the real displacement of the sliding top in contact with the gel was tested by using the INSTRON 4464. The compression tests were performed on the device embedding the alginate gel at a velocity of 5 mm/min. Data were acquired at a frequency of 100 Hz. The experimental data were then compared (Figure 3D) to the data derived from FEM simulations and to data derived from the analytical model, based on Eq. 5.

## Device Testing

### Radiation Force Measurement

The acoustic radiation force generated by a focused ultrasound transducer (16 channels, 20 Watt/channel, Imasonics) was measured by using a radiation force balance (RFB) (International Electrotechnical Commission [Iec], 2006), with an experimental setup as in the Supplementary Figure S3. The focused transducer (1.2 MHz central frequency) was driven by a multi-channel US generator (16 channels, 20 Watt/channel, Image Guided Therapy). A US probe (PA7-4/12, Analogic Ultrasound), placed confocally to the focused ultrasound transducer, was used as a guide for the correct positioning of the transducer focal point. Indeed, the dimensions of the focal spot are around 2–3 mm (Cafarelli et al., 2018), so the US beam can be easily focused on the sliding top (10 × 12.5 mm).

An ABB IRB 200 manipulator was used to move the transducer and the monitoring probe into the correct position.

Sonication parameters (frequency, power, therapy duration, pulse duration, and duty cycle) could be set by exploiting a dedicated interface previously developed (Tognarelli et al., 2017). A digital balance (WLC 20/A2, RADWAG) with 20 kg weighing capacity and 0.1 g resolution, was used to quantify the radiation force.

**FIGURE 4:**
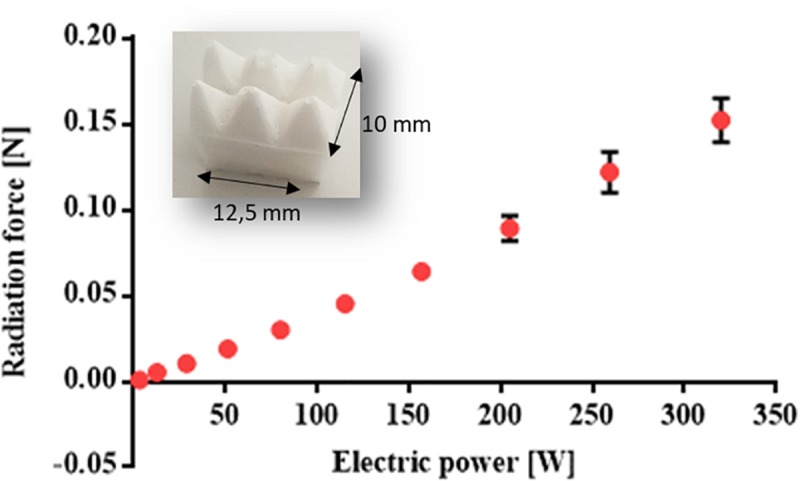
Graph of the measured radiation force [N] at different electric powers (W) (*n* = 3). The picture in the inset shows the nano-doped PDMS layer with a pyramidal structure used to maximize acoustic absorption.

The acoustic power (*P*_*ac*_) applied to the device was calculated as follows:

(6) $$P_{a\,c}=\frac{2\,c\,g\,\Delta \,m}{1+\cos\gamma }$$

where *c* is the speed of sound, *g* is the gravitational acceleration, Δ*m* is the measured difference in weight on the RFB and $\gamma =\sin^{-1}\frac{R_{t}}{R_{c}}$ is the beam convergence angle, in which *R*_*t*_ is the radius of the ultrasonic transducer active element (60 mm in our case) and *R*_*c*_ is the geometrical focal length, i.e., the radius of curvature of the ultrasonic transducer (120 mm in our case). The acoustic radiation force was measured by focusing the US beam on the device top. This was provided, on its surface, with a 1 cm thick polydimethylsiloxane (PDMS) layer with 1:10 monomer/curing agent ratio, doped with 10% w/w BaTiO_3_ nanoparticles (Figure 4). This element has a pyramidal structure to maximize US energy adsorption, thus maximizing the acoustic radiation force on the gel, once integrated into the device. The choice of this specific substrate was based on previous results obtained by the authors on the acoustic properties of bare and nano-doped materials (Cafarelli et al., 2017).

Figure 4 shows the results obtained in terms of radiation force [N] as a function of the electric power [W].

### Drug Delivery Tests

**FIGURE 5:**
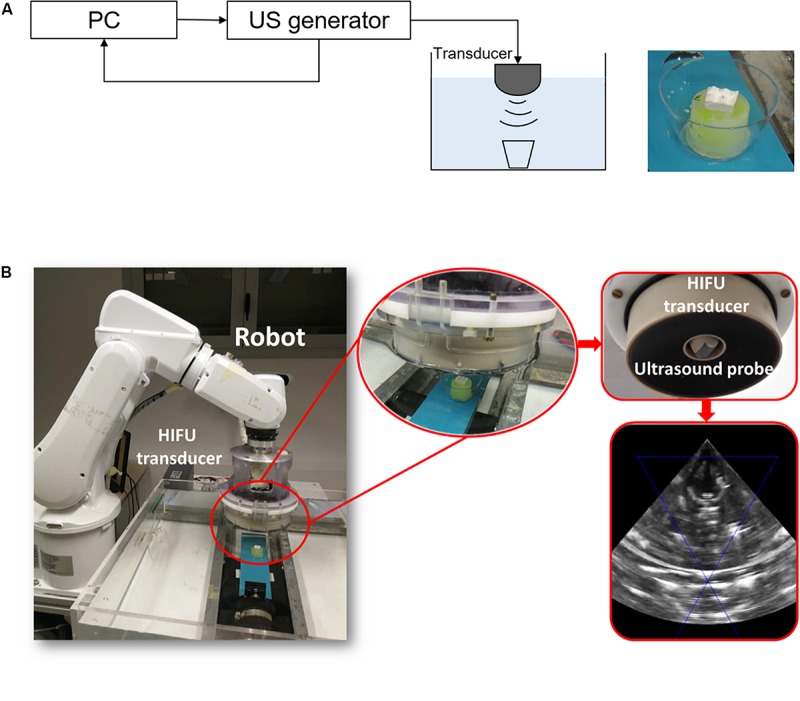
**(A)** Scheme of the set-up used for drug delivery tests. **(B)** Pictures of the experimental set-up with the echographic image acquired by the ultrasound probe.

Delivery tests were performed by using the set-up shown in Figure 5B and on the final assembled device shown in the zoom of Figure 5A (see also Supplementary Movie S2).

The device was placed in a small case (with a volume of 40 mL) completely filled with a 0.9 wt% NaCl, and sealed using a 25 μm thick US transparent polystyrene membrane (Goodfellow), to prevent US attenuation/reflection (Salgarella et al., 2017).

In order to have a pulsatile drug delivery, the device was stimulated by using a pulsed US input. To select the optimal stimulation parameters, different preliminary experiments were performed by varying power and duty cycle of the input wave and by keeping fixed the total duration of the stimulation (3 min) and the pulse repetition period (5 s).

Three different powers (115, 156, and 205 W) and duty cycles (10, 20, and 40%) were tested to evaluate: (i) the temperature increase, which is dependent on both power and duty cycle, and (ii) the displacement of the sliding top, which depends only on the power.

Temperature was measured by using a 50 μm thermocouple (fine wire thermocouple copper-constantan type T, OMEGA) positioned within the device case. Data were acquired through a converter (NI USB-TC01) connected to the computer via a USB port. The acquisition frequency was set to 1 Hz. A thermocouple diameter of 50 μm was selected in order to minimize the interference with the US beam (Hynynen and Edwards, 1989).

**FIGURE 6:**
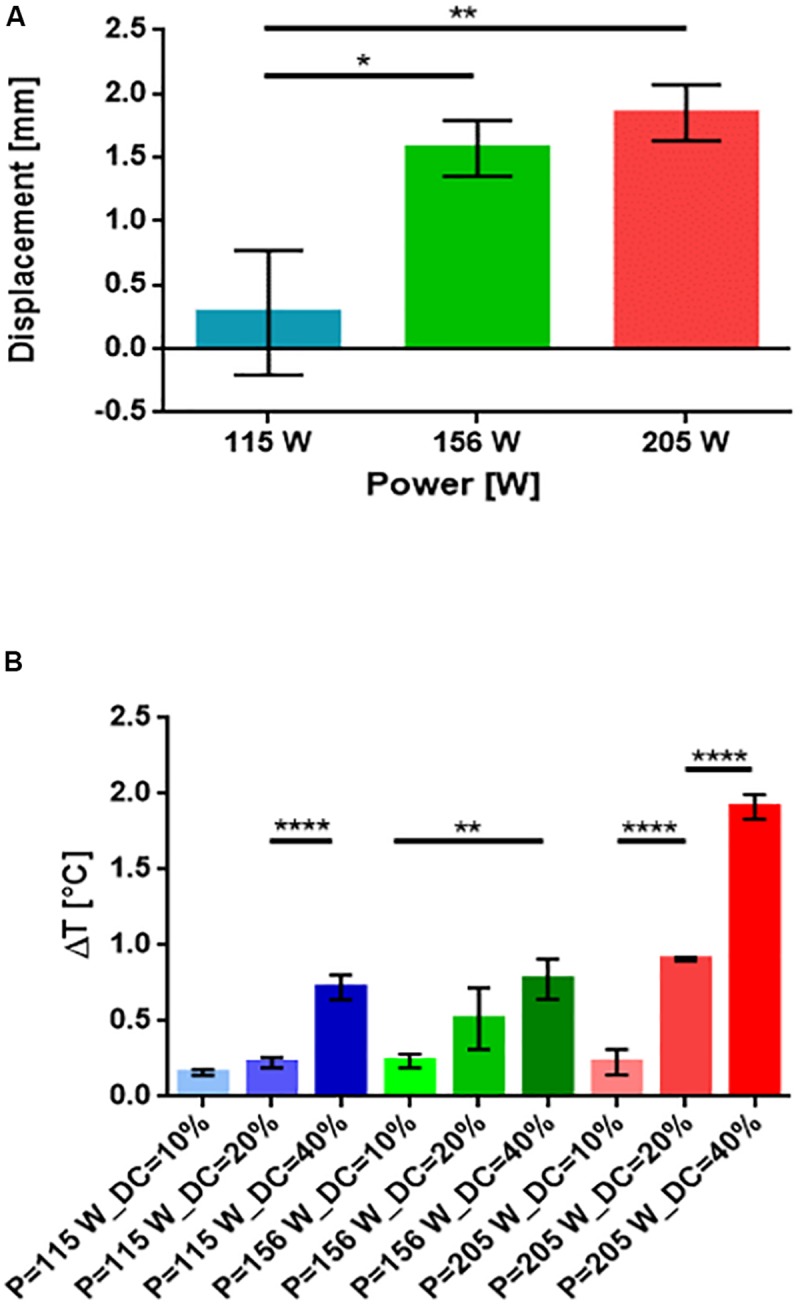
**(A)** Histograms of the displacement of the sliding top in function of three powers (115, 156, and 205 W) (*n* = 3). **(B)** Histograms of the increment of temperature produced by the increment of the duty cycle and of the power (*n* = 3). ^∗^*p* < 0.05, ^∗∗^*p* < 0.01, ^****^p < 0.0001.

To evaluate and quantify the displacement of the sliding top, an analysis of B-mode ecographic images recorded during the stimulation was carried out. The results are illustrated in Figure 6.

All results were reported as mean values ± standard error of the mean. A Holm Sidak test was performed for comparison between two groups. Significance was set at 5%.

Based on the results shown in Figure 6, we selected a specific value of power (205 W) and a specific duty cycle (20%) for the final delivery tests. These values were selected in order to achieve the highest displacement of the sliding top without inducing a potentially dangerous temperature increase: (i.e., slightly below 1°C).

Drug release tests were performed on 10 samples (5 devices were stimulated and 5 used as control). The stimulation was applied for 3 min, with a pulse repetition period of 5 s, a duty cycle of 20%, and a power of 205 Watt. The stimulations were repeated over 2 days with the following protocol: three stimulations in the first day (0, 4, and 8 h) and other two at 24 and 48 h.

Before and after each stimulation, the total volume of liquid was withdrawn from the case in which the device was immersed and 200 μL was transferred to a 96 well plate for fluorescence measurements, performed in triplicate for each sample and for each time-point.

The measured fluorescence intensity was then converted into drug concentration and mass by using a calibration curve previously prepared for fluorescein sodium salt, by imposing different known concentrations.

**FIGURE 7:**
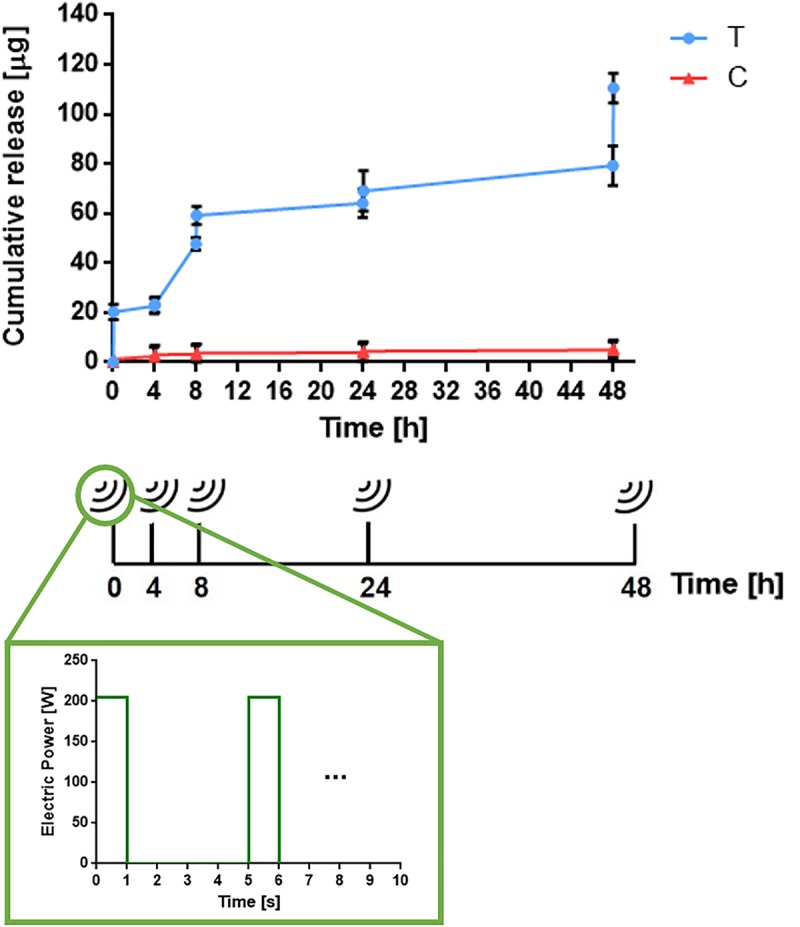
Example of the cumulative drug release from a device triggered by the acoustic radiation force (blue curve) respect to control (non-stimulated) samples (red curve) (*n* = 3). Bottom image shows the stimulation wave applied on the device (*p* = 205 W and DC = 20%). Each pulse series is applied for 3 min.

The results are shown in Figure 7 in terms of cumulative mass release. The system showed a pulsatile drug delivery behavior in correspondence to the stimulation time-points. This demonstrated the possibility to release a significant amount of drug on-demand, with respect to control (non-stimulated) systems, whose release profile always remained close to zero, over the 2 days.

The developed system is a proof of concept of an innovative technology that needs further developments in order to minimize the off-time period releases and to optimize the control of the drug quantity delivered, corresponding to each US stimulation. Currently, as visible in Figure 7, such quantities are not always constant. Such heterogeneity could depend on the fact that the drug is released from the top gel surface, thus creating a concentration gradient in the gel after the first stimulation. However, in view of a possible future optimization and clinical translation of the system, it will be possible to better tune the delivered drug at specific time points. By tuning the US stimulation parameters (i.e., power and duty cycle) it will be possible, for example, to achieve the release of the same quantity of drug at different time points.

### Device Scalability and Alternative Materials

**FIGURE 8:**
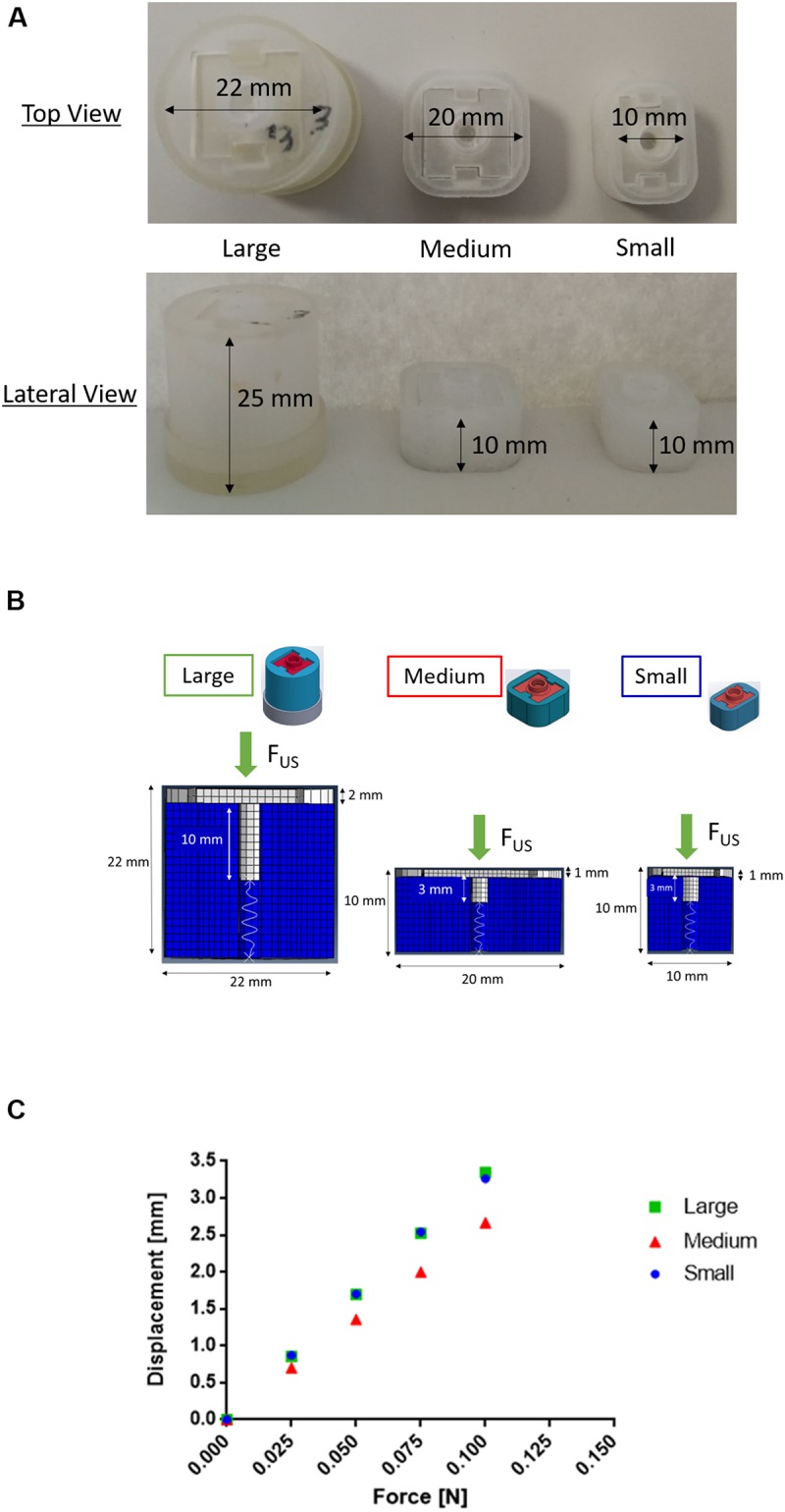
**(A)** Top and lateral views of three prototypes printed in Visijet M3 Crystal having different sizes. The “large” system has a diameter of 25 mm and a height of 22 mm, the “medium” one has a square base of 20 mm and a height 10 mm, while the “small” one has a rectangular base of 10 × 20 mm and a height of 10 mm). **(B)** Comparison between three scaled geometries (here called “large,” “medium,” and “small”) of the PDDS described in the paper. **(C)** Results obtained from FEM simulations in terms of displacement of the sliding top following the application of 0.025, 0.05, 0.075, and 0.1 N. In the smaller device the displacement is equal to the larger one, instead in the medium device it is slightly lower in correspondence to the same forces applied (a displacement of around 3.3 mm in the larger and smaller device and 2.7 mm in the medium one is obtained with a 0.1 N force).

To demonstrate that scaling down in dimension the device does not compromise its working principle, we produced scaled versions of the prototype, defined as “medium” and “small” devices, shown in Figure 8. These systems were modeled by means of FEM simulations, by setting a spring stiffness at 0.02 N/mm and keeping the same settings used in the FEM simulations made for the first prototype (defined as “large,” in Figure 8A). The vertical displacement of the sliding top was evaluated as the main result of the simulation (Figure 8B). By applying the same radiation force (i.e., 0.1 N) perpendicular to the sliding top of the device, the induced displacement of the sliding top in the small device resulted equal to the larger one, while it was slightly smaller for the medium device (Figure 8C). These results are promising in view of a future optimized version of the device, tailorable in terms of dimensions and thus of possible implantation sites.

To demonstrate the possibility to develop the device in a biocompatible material and with a smaller size (thus more suitable for implantation), a device in poly(lactic acid) (PLA) was printed by using a Fused Deposition Modeling 3D printer (see Supplementary Figure S1). The assumption of negligible friction was still valid for the PLA-based device, being similar to the previously described system (see Supplementary Figure S2).

### Validation With Tissue-Mimicking Phantoms

For a more convincing *in vitro* demonstration of the technology, we performed additional tests with the device developed in PLA and by using a more realistic experimental scenario. In particular, we used a 2 cm-thick fat tissue-mimicking phantom made of agar (2% w/v) and aluminum oxide (Al2O3) powder (1% w/v). This composite mimics the acoustic properties of human fat tissue: speed of sound of 1481.6 m/s, acoustic impedance of 1.55 MRayl and attenuation coefficient of 0.65 dB/cm (Gherardini et al., 2017).

**FIGURE 9:**
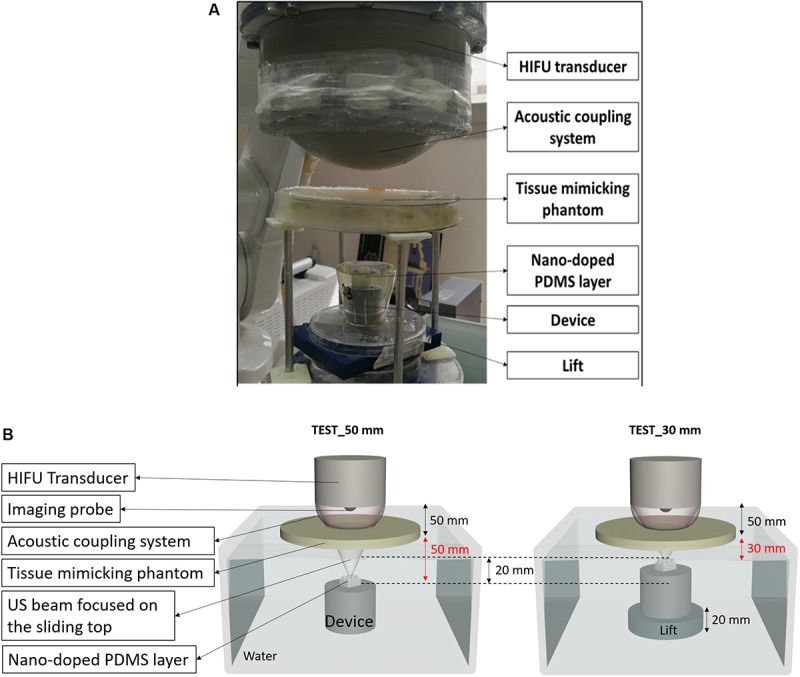
**(A)** Picture of the experimental set-up used to demonstrate the feasibility of the technology in a realistic simulated environment. **(B)** Scheme of the set-up used for the tests; the device was positioned at two different depths (50 mm and 30 mm) from the tissue-mimicking phantom surface.

Tests were performed by using the set-up shown in Figure 9A. To replicate the interface between the focused ultrasound transducer and the patient’s skin, the phantom was located above the water level of a tank filled with deionized and degassed water. The transducer-patient coupling system – which guaranteed a good energy transmission to the target - was composed of a thin latex membrane (150 μm width) filled with deionized and degassed water and fixed to the focused ultrasound transducer.

In order to demonstrate the possibility to target also different points positioned at various depths from the skin patient surface, we performed two experiments depicted in Figure 9B. In these experiments, the target was positioned at 50 and 30 mm, respectively, below the tissue-mimicking phantom. In both cases, the movement of the sliding top was achieved, as shown by real-time ultrasound imaging (Supplementary Movies S3, S4).

It is worth mentioning that the focused ultrasound transducer used in this test was a 120 mm diameter annular phased array transducer (Cafarelli et al., 2018) able to electronically steer the focus of a few cm, from its geometrical natural focus (i.e., 100 mm), along the main axis. Targets at different depths (e.g., very superficial ones) could be also targeted by using focused ultrasound transducers with a different size and geometry. In addition, in order to reach the target *in vivo* with the same acoustic radiation force (*F*_*US*_), the power should be slightly adjusted, taking into account patient-specific acoustic phenomena, such as reflections, diffractions and attenuations caused by natural tissues positioned in the acoustic path. Several acoustic propagation simulation tools [such as e.g., k-Wave acoustic toolbox for Matlab, PZFlex Software and COMSOL Mutiphysics (Treeby and Cox, 2010)] are already widely accepted in the ultrasound scientific community and allow to adapt the exposure parameters to a specific patient anatomy, taking into account all these aspects. As reported in section “Concept and System Design,” in fact, the acoustic radiation force can be defined as shown in equation 1.

In addition, it is worth mentioning that since the beam is focused on the nano-doped PDMS layer put on the sliding top, and not directly on the device, the absorption coefficient (α) is known and is always constant (Cafarelli et al., 2017), even if the device is produced in a different material (i.e., PLA). Overall, these results demonstrate the correct operation of the system also in conditions similar to the *in vivo* ones.

## Discussion

The obtained results constitute an advancement in the field of targeted and remotely controlled drug delivery. Despite previous state-of-the-art reports, the opportunity to exploit acoustic radiation force for biomedical applications, such as manipulation of cells in suspension, increasing the sensitivity of biosensors and immunochemical tests, assessing viscoelastic properties of fluids and biological tissues, elasticity imaging, monitoring ablation during therapy and targeted drug and gene delivery (Lum et al., 2006; Kilroy et al., 2012; Shung, 2015), this paper constitutes one of the really few examples in which this wireless force is used to trigger drug release from an *ad hoc* designed system.

Moncion et al. (2018) have studied focused 2.5 MHz US to sequentially release two fluorescent payloads, each encapsulated within a separate monodispersed perfluorocarbon double emulsion, that are contained within a single acoustic responsive scaffold. The release strategy involved sequential US exposures, whereby the first and second payloads were released at different acoustic pressure regimes. Even if their study presents some similarity with our work (i.e., use of focused US to temporally control the drug release), they demonstrated just a two-shot delivery exploiting acoustic droplet vaporization and cavitation. Differently, we developed an “on-off” device triggerable multiple times by using the radiation force of ultrasound.

Ordeig et al. (2016) developed an implantable capsule exploiting focused ultrasound to reversibly release the encapsulated drug from a thermoresponsive polymer. Here, US was focused on the device and the temperature increase was controlled and kept under the safety threshold (43°C) by using magnetic resonance imaging (MRI). In our case no thermal effects are needed to let the drug exit, being the triggering mechanism a fully mechanical one. This makes the use of complex and expensive systems such as MRI unnecessary. Furthermore, we demonstrated that the temperature increases due to the triggering procedure remaining below 1°C, thus not raising temperature-associated safety issues.

Other studies demonstrated the possibility to have a pulsatile drug release from hydrogels that can be reversibly destroyed by the application of the US stimulus (Huebsch et al., 2014; Huang et al., 2017). With respect to these papers, we used US not to destroy the gel but just to open the device and let the drug exit. Thus, we aimed to increase the pulsatility of the release by minimizing the basal drug diffusion and by adding an on-demand activation degree of freedom of the system, based on the radiation force.

The idea of using compression forces to squeeze a drug-loaded hydrogel embedded in a miniaturized device was proposed by Iacovacci et al. (2015) to enable controlled release of anti-cancer drug after navigation in small caliber body conduits by using magnetic actuation. However, in the mentioned study, only one-shot deliver was possible, due to the non-reversible nature of the trigger (attraction of two small permanent magnets).

As mentioned in the introduction, a PDDS can be useful for the treatment of chronic disorders. An example of such target pathologies is rheumatoid arthritis (RA). RA is a chronic inflammatory autoimmune disorder that causes stiffness, swelling and pain to body joints and typically affects 0.5−1.0% of the population (Köller, 2006). RA has a timed manifestation of its symptoms that is also reflected in the production of proinflammatory cytokines and disease-specific auto-antibodies. In order to have a targeted drug release synchronized with the onset of symptoms, a PDDS could be implanted in the site of interest (i.e., in the knee, the shoulder or the hip) and activated by the user only when needed. To this aim, the system should be designed *ad hoc* for the specific location, application and expected duration/frequency of the therapy.

The use of US technology, in this paradigm, enables a remote control of such a device in the body, in a fully non-invasive manner. After each series of US stimulations, the drug concentration within the hydrogel is balanced in the whole volume, thus to always obtain a relevant quantity of drug available in the top volume of the device, ready to be squeezed out by the following US action. This is supported by previous reports, highlighting a good diffusivity of different drugs [e.g., dextrans (Iskakov et al., 2002), antibiotics (Gordon et al., 1988), theophylline (Grassi et al., 2001), acetaminophen (Aslani and Kennedy, 1996), etc], having a wide range of molecular weights, within alginate gels.

In this study we used only one concentration of the model drug (1 mg/ml), dictated by the solubility of the compound in water. Thus, we did not directly evaluate the encapsulation efficiency nor the loading capacity of our system. Indeed, we assume that the whole amount of drug was successfully encapsulated inside the gel, since we avoided the washing steps which could lead to losses. With this concentration, we observed sufficient amounts of drug released at the desired time-points, compatible with possible clinical applications (e.g., in case of rheumatoid arthritis, doses in the order of micrograms are needed, which were achieved in our case). Since the amount of loaded drug (connected to the loading capacity and encapsulation efficiency) would have an impact on the release kinetics, this should be determined through *ad hoc* measurements on the different drugs used, for each specific system and for each target application.

Once implanted, the device may cause the formation of a fibrotic capsule which in turn may affect drug dosage levels. A possible solution to reduce the fibrotic response is the use of anti-fouling coatings on the device surface. For example, soft hydrogel zwitterionic coatings have been already demonstrated to minimize fibroblast and macrophage adhesion (Trel'ovaì et al., 2018). In addition, it has been demonstrated that *in vitro* models can be used to determine the permeability of fibrous tissues to drugs (Wood et al., 1995). In this study, the transport of three different compounds (with increasing molecular weights) through the implant-generated fibrotic capsule tissue was assessed. Results highlighted that compounds can cross the fibrotic capsule, which is more permeable to molecules featured by a smaller molecular weight. Thus, once known the permeability of the fibrotic capsule to the drug of interest, the fibrotic capsule formation and the consequent change in permeability could be predicted and taken into account, in the therapy design.

## Conclusion

In this paper, a proof of concept of an innovative pulsatile drug delivery system remotely triggered by an externally controlled acoustic radiation force is reported. The acoustic radiation force was used to activate in a fully wireless fashion the sliding of the device top, an event that opened a gap through which the drug contained in an alginate gel was delivered. The device is featured by a directional release (at the top side). Thus, an appropriate position and orientation of the device must be chosen by the physician during the device implantation phase, depending on the anatomical and acoustic constraints of the target area. The drug releasing side will be oriented toward the desired target tissue/region and the US-based activation of the system will be performed by exploiting appropriate acoustic windows.

The stimulation was applied on the device for 3 min with a pulse repetition period of 5 s, a duty cycle of 20% and a power of 205 W. Modulating the US stimulation conditions would enable to explore different drug release ranges, in future evolutions of the device.

These parameters produced a pulsatile drug release behavior that resulted significantly different from non-stimulated controls and that induced a temperature increase smaller than 1°C, thus compatible with future clinical applications.

The device described in this paper could be used to release the drug at the onset of patient’s symptoms, e.g., through a wearable small size system [Tsakalakis and Bourbakis, 2013; Sustained Acoustic Medicine (sam^®^) Pro 2.0 Low Intensity Ultrasound Device (ZetrOZ Systems)^1^; Melmak Ultrasound Device, Biomedical Tissue Technology Pty. Ltd., Sydney, Australia]^2^ able to activate the ultrasound stimulation on-demand, by the patient. In particular, it would be possible to develop an *ad hoc* brace with a special housing for the HIFU transducer. This housing could be properly positioned by using a commercial echography imaging probe in order to find the correct alignment between the US beam focus and the sliding top. This procedure could be performed by the physician once the device is implanted and the treatment could be performed at home by the patient in an autonomous manner. If the target pathology requires a pre-defined delivery of drugs at precise time-points, the system could be provided with a control system activating the transducer at such time-points. For a translation of the technology to the clinic, further efforts will be surely necessary to slightly adjust the US stimulation in order to reach the target with the desired dose. To this purpose, patient-specific acoustic phenomena, such as reflections, diffractions and attenuations due to the natural tissues positioned in the acoustic path, must be carefully taken into account, by using dedicated mathematical US propagation models.

The device proposed in this paper features a slight (but detectable) passive release of drug in the 24–48 h period, which is undesirable for a perfectly controllable pulsed drug delivery system. This is probably due to sub-optimal features of the prototype, such as possible misalignment of the sliding top, excessive permeability of the material used to build the prototype (Visijet M3 Crystal) to the fluorescein sodium salt that may imply a certain release also during the OFF-stimulation phase. Such sub-optimal features should be fixed in the pathway toward clinical translation. Therefore, future evolutions of the device will concern the design of *ad hoc* systems (in terms of device size, shape and materials and of focused ultrasound transducers) depending on the specific target disease and site of implant.

## Data Availability Statement

The datasets generated for this study are available on request to the corresponding author.

## Author Contributions

LR and AC conceived and designed the study. SC was the main operator of the experiments. SC and AZ synthesized and characterized the hydrogel and conducted *in vitro* drug delivery tests. SC and AC designed the experimental setup and performed radiation force measurements and drug delivery tests. SC performed FEM simulations and data analysis. LR supervised the research group. SC wrote the manuscript. All authors reviewed the manuscript.

## Conflict of Interest

The authors declare that the research was conducted in the absence of any commercial or financial relationships that could be construed as a potential conflict of interest.

## Footnotes

1 https://zetroz.com/sam-pro-2-0/

2 https://stimulatehealth.ca/bone-stimulators.php
