# Supplementary figures and images for: Pulsatile Drug Delivery System Triggered by Acoustic Radiation Force
Source: Front Bioeng Biotechnol. 2020 Apr 17;8:317. doi: 10.3389/fbioe.2020.00317 (PMC7202567; doi:10.3389/fbioe.2020.00317)

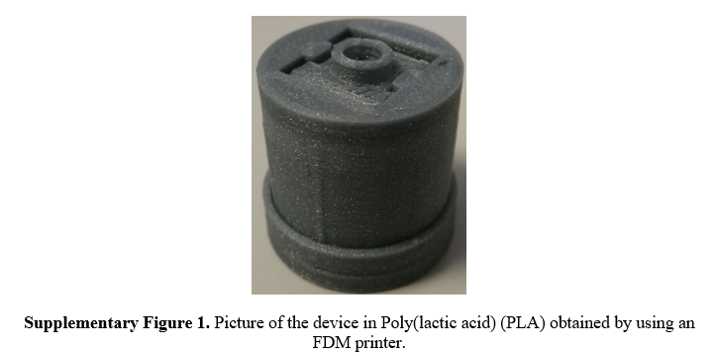

Supplement: Supplementary file 1 [file Image_1.tif]

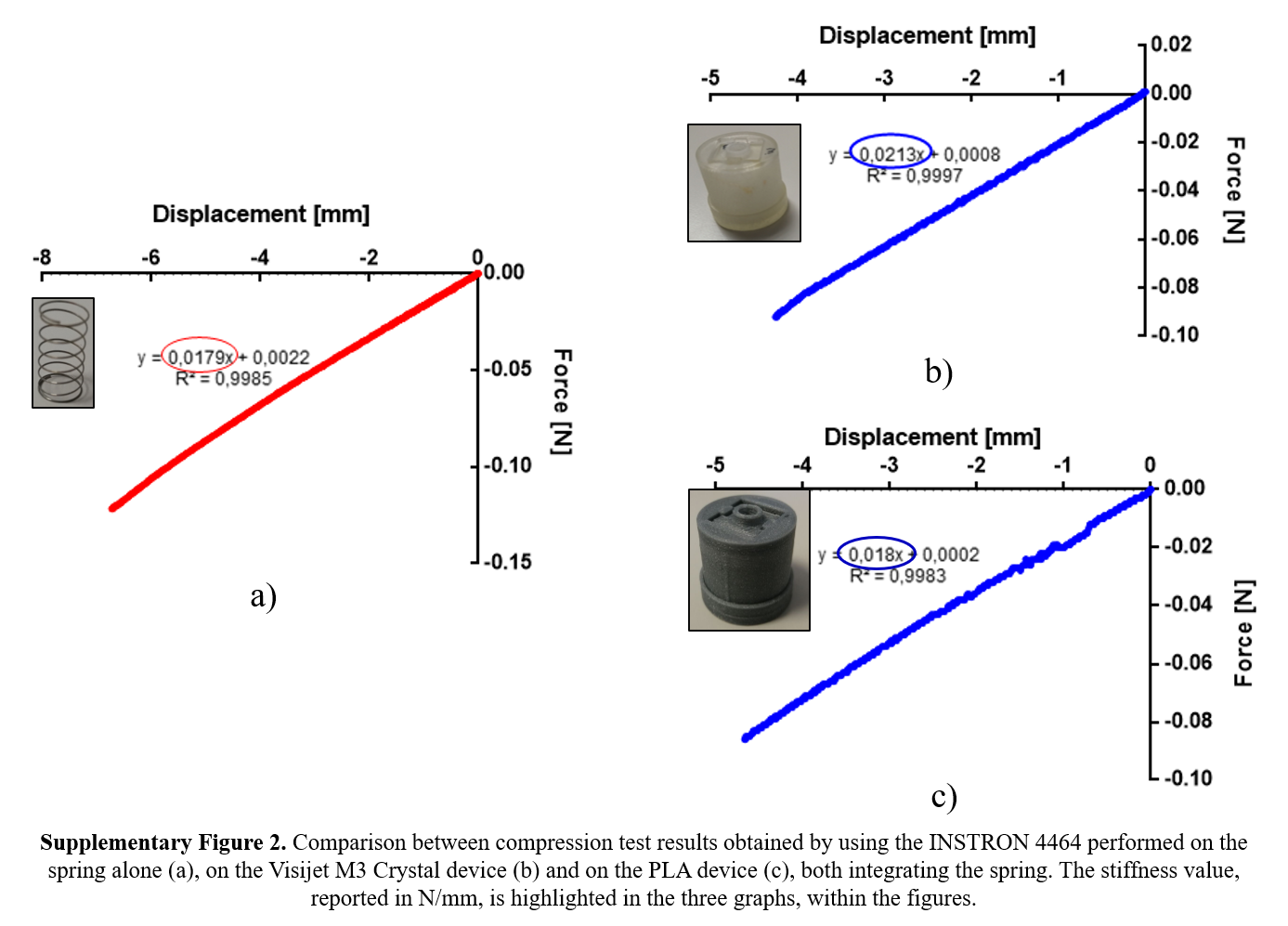

Supplement: Supplementary file 2 [file Image_2.tif]

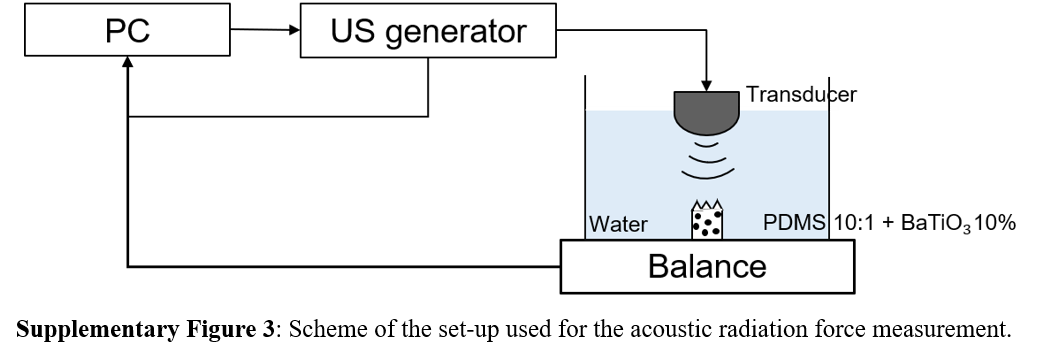

Supplement: Supplementary file 3 [file Image_3.tif]
